# Recollections on Sixty Years of NBS Ionizing Radiation Programs for Energetic X Rays and Electrons^1^

**DOI:** 10.6028/jres.111.035

**Published:** 2006-12-01

**Authors:** H. William Koch

**Affiliations:** University of Denver, Denver, Colorado 80208

**Keywords:** absorbed dose, betatron, CIRMS, electrons, ionizing radiation, rad, radiation processing, radiation protection, reference data, synchrotron radiation, x rays

## Abstract

These recollections are on ionizing radiation programs at the National Bureau of Standards (NBS) that started in 1928 and ended in 1988 when NBS became the National Institute of Standards and Technology (NIST). The independent Council on Ionizing Radiation Measurements and Standards (CIRMS) was formed in 1992. This article focuses on how measurements and standards for x rays, gamma rays, and electrons with energies above 1 MeV began at NBS and how they progressed. It also suggests how the radiation processors of materials and foods, the medical radiographic and radiological industries, and the radiological protection interests of the government (including homeland security) represented in CIRMS can benefit from NIST programs.

## 1. Introduction

These recollections are on ionizing radiation programs at the National Bureau of Standards (NBS) that started in 1928 [1,2] and ended in 1988 when NBS became the National Institute of Standards and Technology (NIST) [3]. The Council on Ionizing Radiation Measurements and Standards (CIRMS) organized by scientists in the fields of industrial radiation processing, medical radiation, the radiation protection of people, and the environment, was formed four years later [4]. Since 1992, NIST and CIRMS have been working together with great effectiveness in advancing current ionizing radiation programs, both at and external to NIST.

Radiation that is ionizing is either particle radiation or electromagnetic radiation in which an individual particle or photon has enough energy to ionize an atom or molecule by completely removing an electron from its orbit. Ionizing radiation includes electrons, x rays, gamma rays, alpha particles, beta particles, and neutrons. These radiations are used extensively in the medical radiographic and radiological industries and the radiation processing of plastics, pharmaceuticals, and foods. They are involved in the radiological protection of the public, including homeland security.

Current work with ionizing radiation at NIST can be reviewed via Internet in such documents as a 2002 Newsletter of the Technical Activities of the “Ionizing Radiation Division” [5,6]. The first page of the Newsletter states the goal of the Division and the strategy for meeting this goal. The goal is “to provide the foundation of ionizing radiation measurements for our nation”. The strategy for meeting this goal “is to develop, maintain, and disseminate the national standards for ionizing radiation and radioactivity to meet national needs for health care, U.S. industry, and homeland security” [5].

The Newsletter also states the three strategic focus areas:

First - Radioactivity Standards;
Second - Neutron Standards and Measurements;
Third - Radiation Dosimetry Standards.

These comprehensive goals and strategies have a long history. Part of that history is summarized in this article by identifying some of the pivotal people, programs, and radiation sources with x rays, gamma rays, and electrons during the start-up of work of the Optics Division and later the Radiation Physics Division (RPD)^2^ at energies below 1 MeV from 1928 to 1945 and above 1 MeV from 1945 to 1988.

Chemical dosimetry and other standards and measurements for radioactivity, neutrons, and nuclear physics are not included but could be the subject of reviews similar to this one at a later date.

The years from 1928 to 1988 include the years immediately after World War II from 1945 to 1962, which have been called by some the “Golden Years of Physics” because of a grateful Congress and a nation willing to fund most sound physics proposals in the U.S. They suggest an appropriate division of the time covered here into three periods: Period 1: 1928 to 1945, when start-up and many contributions to radiation shielding and personnel protection at energies below 1 MeV were made by the staff of RPD and the National Council on Radiation Protection and Measurement (NCRP), both located at NBS in Washington, D.C.; Period 2: 1945 to 1962, when two high-energy betatrons were added to seven other radiation sources for research in Washington; and Period 3: 1962 to 1988, when staff, three new electron accelerators, and most other radiation sources at NBS were relocated to new facilities in Gaithersburg, Maryland and when NCRP was relocated to Bethesda, Maryland. These recollections end by stating the need for the founding of CIRMS.

## 2. Period 1: 1928 to 1945

At the beginning of Period 1, Lauriston S. Taylor, 28 years old, was hired at NBS as a physicist to measure absorbed dosages in roentgen units produced by x rays and radium gamma rays in tissue-like and other materials. Taylor soon became Chairman of NCRP. Note: A chronology of leadership assignments at NBS in RPD is given in Appendix A [1,2].

There followed fifteen years during Period 1 and seventeen years in Period 2 of active experimental research and theory of measuring instruments and shielding of personnel against radiations. Those radiations were produced by radioactivity sources and electron and proton accelerators with voltages up to 3 million volts but with low beam intensities. Taylor coordinated NCRP committees of scientists and engineers who studied and recommended guidelines that were subsequently published in internationally recognized reports and handbooks. A list of those NCRP reports and handbooks issued during the time period 1928 to 1989 is given in Appendix B [7].

The coverage of radiation types and energies in Appendix B, as well as of the problem areas and situations, is comprehensive. When the high regard of the science and engineering community for these reports issued by NCRP is also appreciated, one can understand the considerable national contributions that were made possible by the technical and organizational skills of Lauriston S. Taylor. A measure of Taylor’s contributions can be obtained from the large number, 1.8 million copies of NCRP handbooks and other formal reports distributed since 1931 [7]. He pursued his work for more than 50 years with the help of large numbers of talented individuals, many of whom volunteered their services.

As stated on the NCRP web site, “the NCRP was reorganized and chartered by the U.S. Congress in 1964 as the National Council on Radiation Protection and Measurements” [7] and was relocated from NBS, first to District of Columbia NW, and then to NCRP offices in Bethesda, Maryland.

Near the end of the first time period, Lyman J. Briggs, the third Director of NBS since its founding in 1900, celebrated his 71st birthday. He submitted his resignation to Secretary of Commerce Wallace on June 25, 1945. Edward U. Condon^3^,^4^, a first-rate theoretical physicist, was recommended as Briggs’ replacement. He was formally appointed NBS Director on November 7, 1945, three months after the dropping of the Nagasaki atomic bomb and the end of the War with Japan to which he had contributed importantly.^5^

## 3. Period 2: 1945 to 1962

In the second period (1945 to 1962), Condon’s attentions initially were directed at administrative, personnel, and budgetary matters but were always focused on need for new, modern, and fundamental research programs. His general goal was to modernize NBS.

Condon’s administrative goals were to bring “the new physics to the Bureau” [9] initially by reorganizing the Atomic Physics, Radioactivity and X rays Sections of the Optics Division into the Atomic and Radiation Physics Division with Taylor as Chief. Taylor’s Division was divided into two laboratories: the Atomic Physics Laboratory (APL) and the Radiation Physics Laboratory (RPL) (Appendix A) [9].

Condon’s personnel goals included the staffing of these laboratories not only with experimentalists, but also with theorists. In 1946, a theoretical physicist, Ugo Fano, who had worked with Enrico Fermi, “was hired to work on x ray photons, and did so brilliantly — on x rays and many other problems. As near as can be determined, this was the first time in its history that the Bureau had hired a pure theorist. He was not to be the last. For the Bureau, a new approach to the conduct of science had begun” [10].

Fano was an excellent choice for the two laboratories, because he was equally well grounded in atomic as well as radiation physics and he worked well with experimentalists. One example of his many contributions to the Radiation Physics Laboratory was his 1959 magnum opus with L.V. Spencer and M. J. Berger entitled “Penetration and Diffusion of X rays” [11]. This had been preceded by another seminal article by Spencer in 1955 entitled “Theory of Electron Penetration” [12]. Other outstanding members of Fano’s theory group included Michael Danos, Leonard Maximon, Sidney Meshkov, and Norwegian Guest Researcher Haakon Olsen.

Condon’s budgetary goals were substantial, but achievable. One result was a commitment by the Bureau, upon the recommendation of Taylor, to purchase not only a 50 MeV betatron, but also a 100 MeV betatron from the General Electric Company (G.E.)^6^ in 1948 [13]. Two betatrons were a major scoop for NBS since betatrons were first developed only eight years earlier at the University of Illinois (U.I.) by Donald Kerst. Before this development, research with x rays and electrons above 1 MeV from accelerators was not available. Only about twelve betatrons had been built at G.E. and the Allis-Chalmers Co. for Los Alamos, arsenals and hospitals, as well as one or two for fundamental research at universities.

Condon and Taylor sought a physicist with some betatron experience to head a Betatron Section in 1949 within the Radiation Physics Laboratory using the two G.E. betatrons. A research assistant professor of physics at U.I., H. William Koch, had been involved in building and operating betatrons from 1941 until 1949 when NBS made Koch an offer. He accepted and became another 28-year-old physicist to head a new Section, as Taylor had done 20 years earlier.

The Section was renamed “High Energy Radiation Section” in the 1950s. Others from Illinois soon were added to the staff of the Section. These included John McElhinney, James Leiss, Everett Fuller, Samuel Penner, Jack Lightbody, Jr., and James O’Connell. Marshall Cleland from Washington University in St. Louis was also one of those early recruits. After a decade of facility development, this Section made a well-rounded combination of basic and mission-oriented research possible, both in the Atomic Physics Laboratory and in the Radiation Physics Laboratory [9].

The APL contributions resulted from the conversion of the 100 MeV betatron to a 180 MeV synchrotron by the General Electric Company in about 1952. This made an accelerator that could generate a strong beam of synchrotron light tangential to the synchrotron electron beam in a vacuum with a large aperture, a capability that became uniquely important to atomic physics research. Robert Madden of the NBS Atomic Physics Division used this light source for his group’s research starting in 1961. It was the first synchrotron to use synchrotron radiation for experimental purposes on a regular basis. The facility came to be known as the Synchrotron Ultraviolet Radiation Facility (SURF) [14].

The initial RPL contributions were reviews of the science that had been used by Taylor and associates since he joined NBS in 1928. Two of the early review reports were “A Theory of Cavity Ionization” by Spencer and Attix in 1955 [15] and “Cavity Ionization Chambers for Radiation Measurements in Food Processing” by Wyckoff and Koch in 1957 [16].

Although the latter report was directed at specialized audiences of potential food processors, it reviewed one of the principal measurement procedures used in the previous twenty-five years at NBS and elsewhere. This procedure used cavity ionization chambers calibrated in roentgen units to measure both the radiation intensity incident on a material as well as the energy absorbed in the material. The number of roentgens is equal to the number of electrostatic units of charge produced in the air of a cavity ionization chamber by the electrons generated by x ray or gamma ray photons per 0.001293 grams of air.

Use of the number of roentgens measured in a cavity placed in the direct radiation beam has made possible measurements relative to the control settings on a given radiation source. The personnel controlling the source must be assured that the radiation source is providing a reproducible photon intensity (i.e., energy per unit time crossing a unit area perpendicular to the radiation direction in units of ergs per square centimeter per sec).

**Fig. 1:**
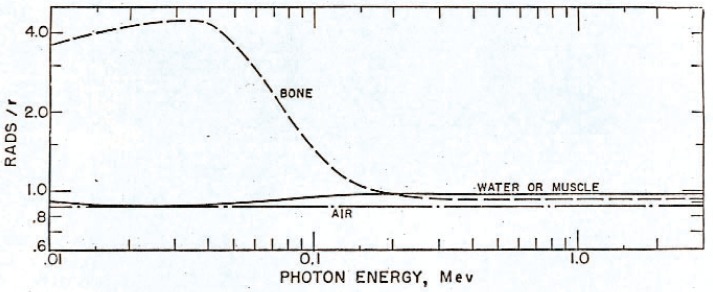
Absorbed dose in rads produced by one roentgen (r) measured in an air cavity in bone, and water or muscle absorbers. Note: the current symbol for roentgen is R.

Use of the number of roentgens (R) to define the absolute values as well as relative values of energy absorbed per unit mass (i.e., rads or 100 ergs per gram) within irradiated materials can often be of critical importance. The calculated ratios of the rads to roentgens respectively for different biological materials and water do not vary appreciably in the energy range from approximately 60 keV to 2000 keV, except in bone, as shown in Fig. 1 [16]. However, because of such problems as bone influencing absorbed dose measurements in medical radiology, the roentgen has been recognized to be an interim measurement procedure until a more accurate system for absorbed dose could be developed.

As will be shown later in this report, a much improved procedure for absorbed dose was later developed in RPL by Steve Domen using a calorimeter for measuring absorbed doses directly in water, a close approximation to tissue. The statistical uncertainties in the absolute values of rads, which are experimental standard deviations of the mean, are of the order of 0.5 % [17]. Domen’s achievement in water calorimetry was preceded by years of graphite calorimetry at NBS and other laboratories with uncertainties above one percent [18].

Another report needed for the interpretation of experiments performed with x ray photons reviewed the bremsstrahlung process for producing x rays in constant-potential accelerators as well as in betatron and synchrotron accelerators of the RPL. Untangling the effects of a spectrum of x rays to obtain the effects produced by photons with a fixed energy was one of the major challenges of research and applications with betatrons. An experimental and theoretical analysis entitled “Bremsstrahlung Cross-section Formulas and Related Data” by Koch and Motz was published in 1959 [19]. This review was and still is used as a reference source for the interpretation of experimental results obtained with spectra of x rays in high-energy electron accelerator experiments.

**Fig. 2:**
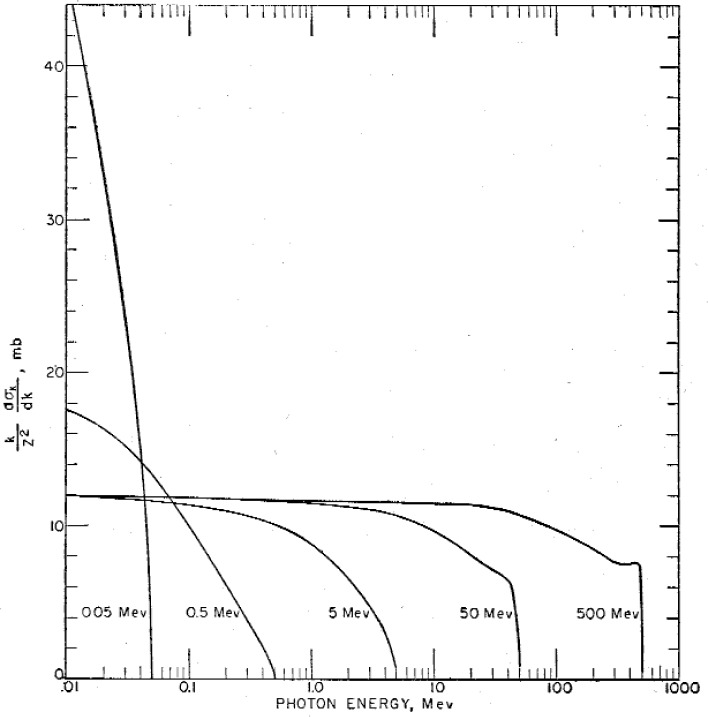
Dependence of the Born-approximation cross section integrated over the photon directions in millibarns (mb) on the photon and electron energy. The maximum photon energy in each bremsstrahlung continuum is equal to the electron kinetic energy noted on each curve producing the bremsstrahlung (e.g., 0.05, 0.5, 5, 50, and 500 Mev).

Figure 2 from the bremsstrahlung article [19] shows a plot of the type of x ray spectra encountered in work with bremsstrahlung where a continuum of x ray photon energies has a maximum photon energy equal to the kinetic energy of the electrons producing the photons.

A process that is the inverse of bremsstrahlung is pair production. This process was also reviewed in an article entitled “Pair Production By Photons” by Motz, Olsen, and Koch and published in 1969 [20]. A related theoretical review was an article entitled “Electron Scattering without Atomic or Nuclear Excitation” by Motz, Olsen, and Koch in 1964 [21].

An example of the measurements that were made with total absorption spectrometers showed the bremsstrahlung challenge clearly. The spectrometer consisted of a large sodium-iodide crystal that was used to produce a scintillation when all or part of the energy of an x ray photon was absorbed in the crystal [22]. Because the crystal was not a large enough cylinder with its 9 inch diameter and 6.5 inch length to make it totally absorbing for the energy of x ray photons of interest (up to 90 MeV) and their by-products, it had poor energy resolution but high detection efficiency. An improvement in resolution was made with two crystals in which a coincidence requirement between two crystals enabled detection of those x ray photons that produced an electron-positron pair near the surface of the large sodium-iodide scintillation spectrometer [23].

**Fig. 3:**
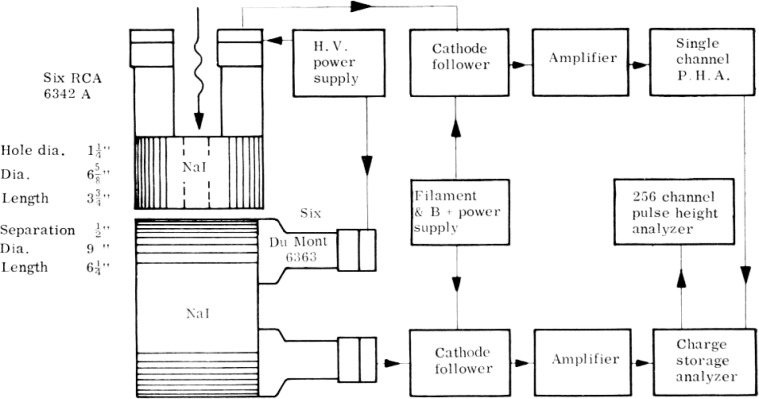
A simplified block diagram of the scintillation pair spectrometer.

**Fig. 4:**
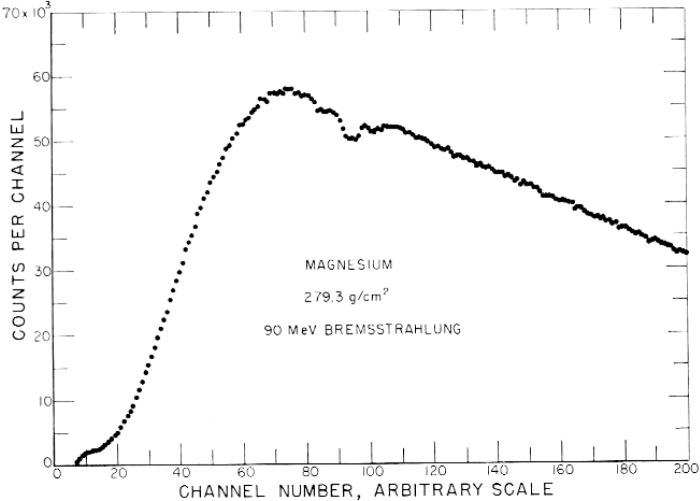
Pulse height distribution obtained with spectrometer in Fig. 3. Channel 200 corresponds to approximately 40 MeV photon energy. Absorber was magnesium.

A block diagram of the two-crystal spectrometer is shown in Fig. 3 [23]. The pulse height distribution shown in Fig. 4 was obtained with the spectrometer detecting 90 MeV peak synchrotron x rays transmitted by a magnesium absorber [23]. Although the dips in the giant-resonance nuclear absorption between channels 80 and 110 in the figure are relatively large, structures in the dip regions were not adequately resolved. Not only was the photon-energy resolution inadequate for accurate nuclear and electromagnetic cross section research, the interpretation of the data was difficult. It was made difficult because the smoothly varying background at all channels and particularly below channels 80 and above 110 resulted from the convolution of the bremsstrahlung spectrum from the synchrotron, the electronic and nuclear attenuation processes in the absorbers, and the response function of the spectrometer.

The resolution of the two crystal spectrometer was reported to be 2.0 ± 0.2 %, which was at least five times better than the single crystal result [23]. However, since the 2 % value is determined in the scintillation pair spectrometer “by the statistical fluctuation of the photomultiplier photoelectrons, it seems unlikely that an improvement is feasible with a scintillation spectrometer. Therefore, for higher resolution spectroscopy a magnetic pair or Compton spectrometer with their small detection efficiency are still required” [23].

An even better approach for some nuclear and electron research is to work with inverse processes in studies of elastic and inelastic scattering of electrons as will be mentioned in the next section. Such equipment was developed at NBS with a high-resolution, magnetic spectrometer by Penner, Lightbody, and Fivozinsky [24,25,26] using electrons from a new NBS electron accelerator.

The work of the National Committee on Radiation Protection and Measurements led by Lauriston Taylor was assisted by members of the High Energy Radiation Section in NCRP Handbooks and Reports listed in Appendix B. Three important handbooks were: “Protection Against Betatron-Synchrotron Radiations up to 100 Million Electron Volts (1954)”, “Shielding for High Energy Electron Accelerator Installations (1964)”, and “Dosimetry of X ray and Gamma Ray Beams for Radiation Therapy in the Energy Range 10 keV to 50 MeV (1981)”. Such contributions supplemented the pioneering work of Taylor, Wyckoff, Motz, Fano, Spencer, and Berger at energies below 1 MeV.

The success of the program with low and high energy x rays was acknowledged by the NBS administration to Lauriston Taylor and staff when he left the Radiation Physics Division in 1962. He was appointed Associate Director for Technical Support of NBS to make possible the redefinition of the role of NCRP. In December he retired from NBS to become head of the congressionally-chartered NCRP. Koch was named the head of the NBS Division in 1962 (see Appendix A).

## 4. Period 3: 1962 to 1988

1962 was a critical time for NBS and the Division. A move of the NBS to Gaithersburg had been announced in 1960. This major move required a lot of planning of facilities, building spaces and program issues. One of the biggest issues was the deficiency at NBS of new, powerful electron-beam sources, then available and being used in U. S. industries. The low source strengths had been regarded as adequate for work in radiation metrology and radiation protection of humans for 30 years. For example, the NBS betatron and synchrotron were high-energy, circular-orbit accelerators that had time-averaged x ray beam powers that were low and measurable in milliwatts. However, many of the applications by industry to materials processing required high beam powers in the 100 kW to 1000 kW range.

Taylor had encouraged the planning by the High Energy Radiation Section of a linear electron accelerator (Linac) with maximum energies equivalent to the NBS circular accelerators, the 50 MeV betatron and 180 Mev synchrotron, but with much higher electron-beam powers. The other sections of the RPD were also encouraged to up-grade their electron accelerators. Thus was developed the new laboratory plan for a traveling-wave L-Band Linac that could produce 50 kilowatts of beam power at 100 MeV with peak energies of 140 MeV at lower power levels. Two other accelerators — a 1.5 MeV electron Dynamitron with 15 kilowatts of beam power and a 4 MeV electron Van de Graaff with 4 kilowatts — were also included.

The entire Division participated in the planning of a large building to accommodate the new sources as well as most of the radiation sources then operating in the NBS Washington laboratories. Of the original two large accelerators obtained from G.E., the decision was made to surplus the 50 MeV betatron and move only the 180 MeV synchrotron. James Leiss supervised much of this planning as well as the Linac installation and operation.

As a result of the planning and architectural design, the new Radiation Physics Laboratory building at Gaithersburg (Building 245) was constructed with a total of 77,000 square feet of useable space. Seventy percent of that space was located below ground level in order to take advantage of the shielding by the ground.

Details of the new facilities were assembled by the NBS public information staff with photographs and discussions with Laboratory staff. The results were printed in 1966 in a booklet entitled: “NBS Radiation Physics Laboratory” [27]. Nine radiation sources were moved from Washington, DC. The Linac and Dynamitron were moved directly from their manufacturers. The final list of RPL and APL facilities at Gaithersburg in Building 245 is given in Table 1 [27].

**Fig. 5:**
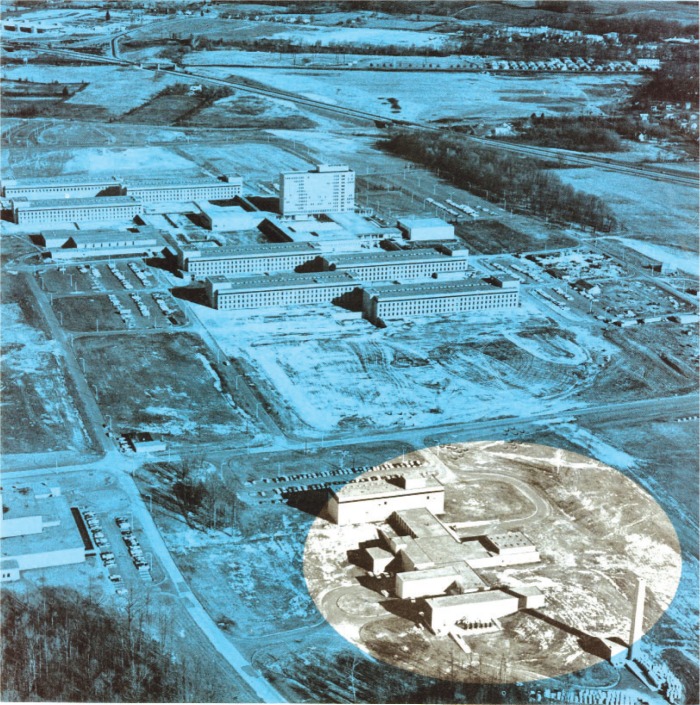
1966 aerial view of the NIST Gaithersburg site with the tall Administration Building in the background and the Radiation Physics Laboratory high-lighted in the foreground.

**Fig. 6:**
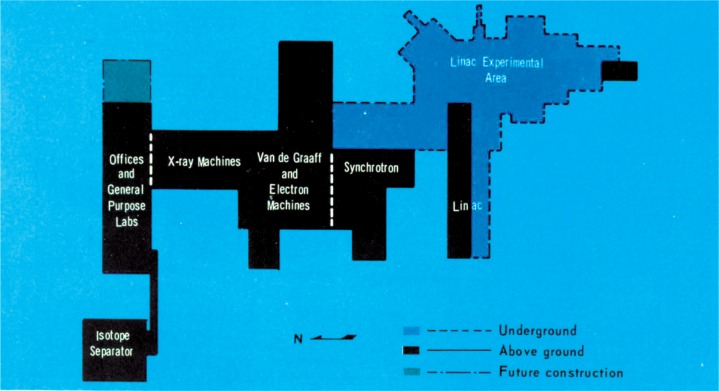
Plan view of the Radiation Physics Laboratory showing above ground and underground construction.

**Fig. 7:**
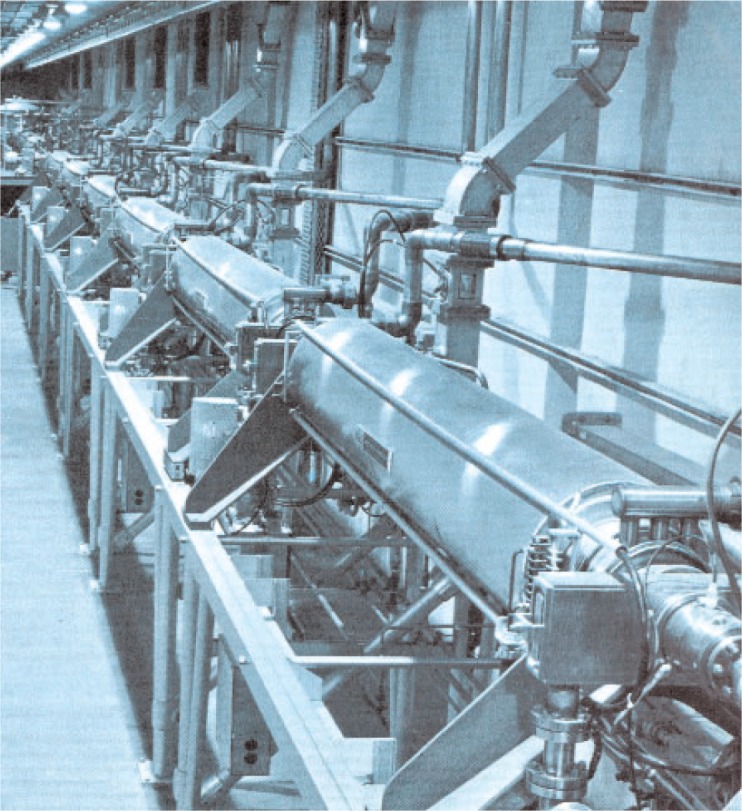
The NBS linear accelerator in place in RPL. This L-Band Linac could produce 50 kilowatts of power at 100 Mev with peak energies of 140 Mev.

**Fig. 8:**
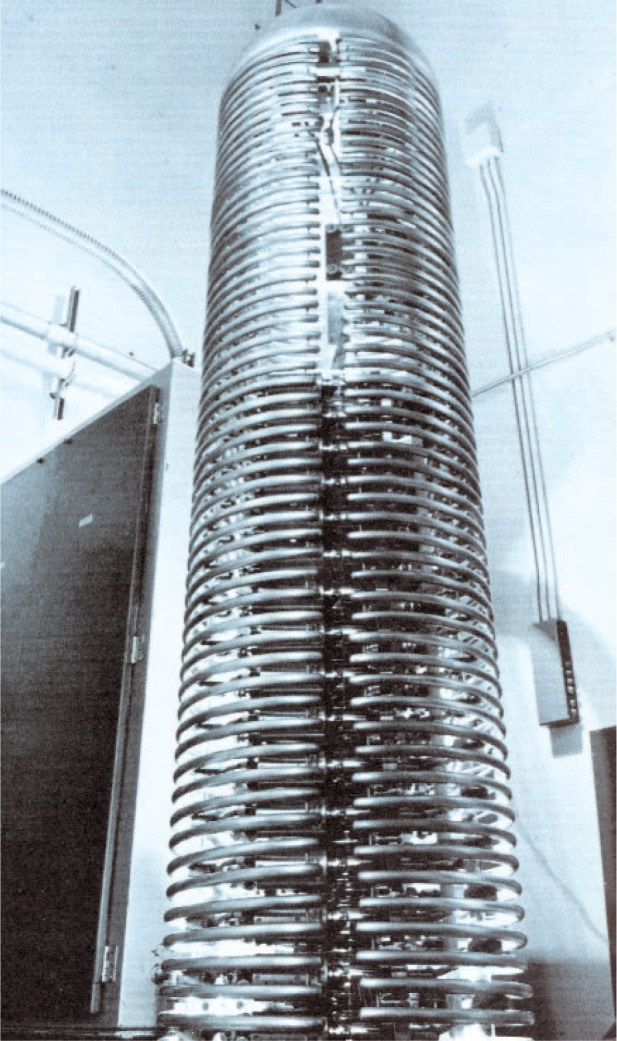
The 1.5 Mev electron Dynamitron that could produce 15 kilowatts of beam power.

**Fig. 9:**
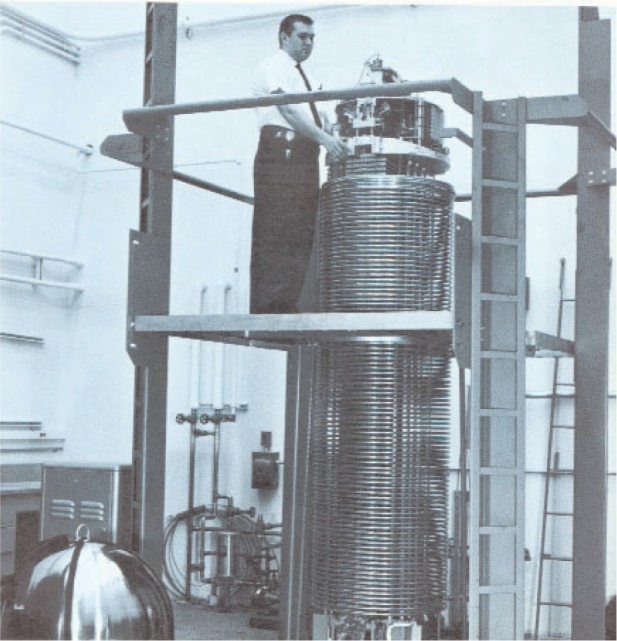
The 4 Mev electron Van de Graaff that could produce 4 kilowatts of beam power.

**Fig. 10:**
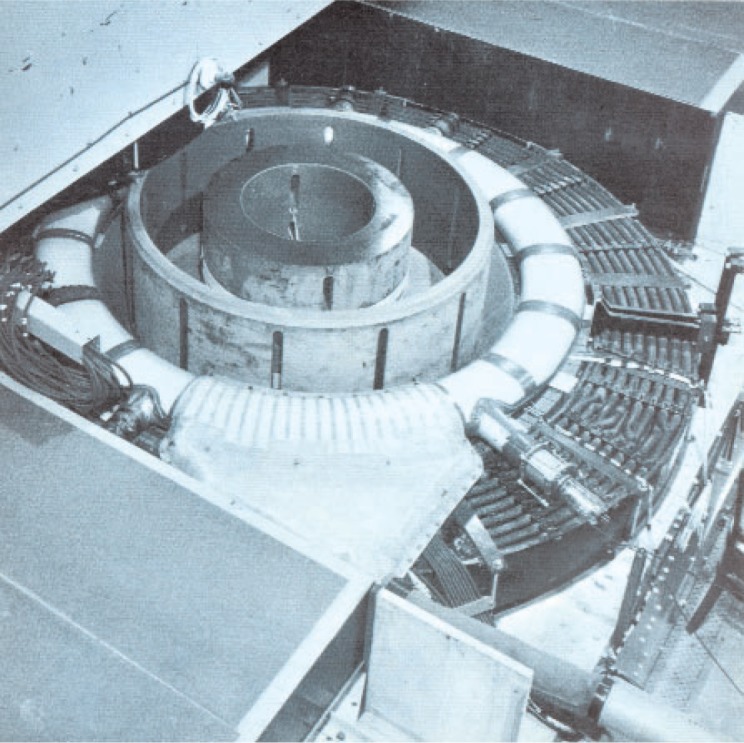
The 180 Mev electron synchrotron with a beam power estimated to be 100 milliwatts.

A few photographs from the booklet show this attractive and unique facility. Figure 5 is an aerial view of the NIST Gaithersburg site with the Administration building in the background and the RPL building in the foreground; Fig. 6 is a plan view of the RPL; Fig. 7 is a photo of the 140 MeV linear accelerator; Fig. 8 is a photo of the 1.5 MeV Dynamitron; Fig. 9 is a photo of the 4 MeV Van de Graaff; and Fig. 10 is a photo of the 180 MeV synchrotron. All three new electron facilities and the older electron and x ray sources listed in Table 1 were operational soon after 1966.

**Fig. 11:**
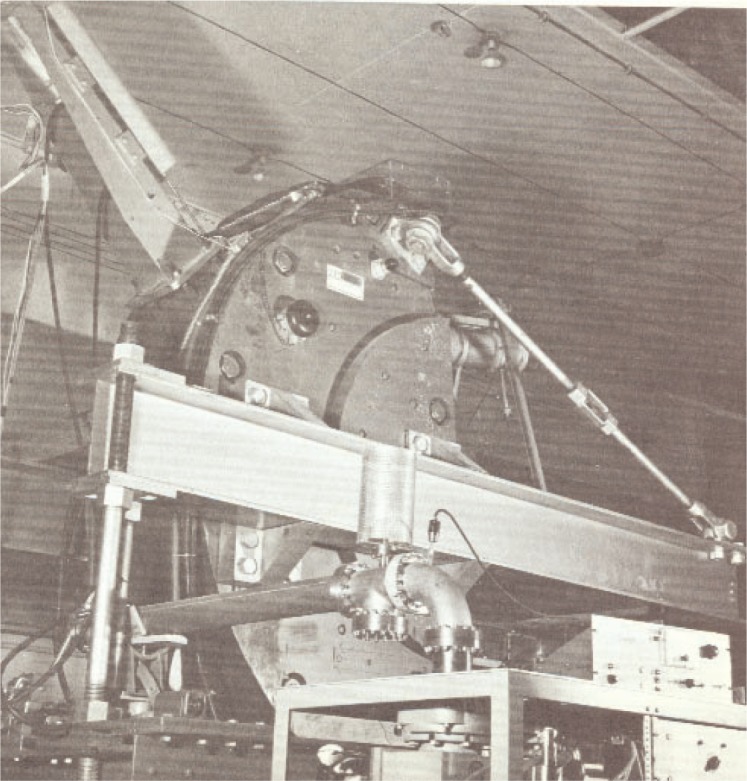
The high-resolution magnetic spectrometer for electron scattering research.

The next major project for the Linac facility was the design and fabrication of the NBS electron scattering spectrometer, shown in Fig. 11, by Penner, Lightbody, and Fivozinsky [25,26]. Penner wrote a description of the spectrometer in an NBS Technical Note entitled “Experimental Techniques for Electron Scattering Investigations” [25]. Required also was a 12-channel semi-conductor counter system for the spectrometer [26]. The spectrometer and counter system were needed to define accurately the energy spectrum of electrons scattered from targets placed in the Linac electron beam.

At the same time in 1966 that the booklet on facilities [27] was completed, Koch and staff undertook to summarize what was known about the applications of high energy electrons. The results were published in a 1967 Science magazine article entitled “Electron Beams: National Bureau of Standards and the New Technology” [28]. Both the booklet and the Science article were intended to announce to the science community generally and the radiation industry particularly the new capabilities of NBS and its willingness to collaborate with U.S. scientists by means of guest worker programs and other joint efforts.

At about this time Koch was appointed Director of the American Institute of Physics, then headquartered in New York City, his home town. Koch left NBS on December 27, 1966. In 1967 Randall Caswell was named Acting Chief of the Radiation Physics Division, and James Leiss was named Chief of the Accelerator Branch of the Division. Leiss was later named the Chief of the Linac Radiation Division in 1968 [see Appendix A].

The Linac and the other facilities were productively used by staff and many guest workers after the move to Gaithersburg and until the end of Period 3 in 1988.

## 5. Examples of Research Highlights

These personal recollections focused on the chronology and events that resulted in the facility, staff and their research programs. This focus was an attempt to capture the spirit and productivity of the staff, and the problems and solutions in research at the forefront of work with electron and x ray radiations produced by radiation sources with beams of high-power. Overall, the Radiation Physics Laboratory at Gaithersburg has proved to be a unique and outstanding national facility for theoretical and experimental research and standards with ionizing radiation.

Specific examples of publications are presented below to illustrate the high quality of the research in this laboratory at NBS Gaithersburg from 1962 to 1988. The publications emphasize radiations with energy above 1 MeV. Research on chemical dosimeters, neutron physics, nuclear physics, and radiations below 1 MeV were equally important but were not considered here.

### 5.1 Theory

With his 1963 paper [29], entitled “Monte Carlo Calculation of the Penetration and Diffusion of Fast Charged Particles”, theorist Martin Berger “quite literally established the Monte Carlo calculation of charged-particle transport at the energies of interest in medical and radiation-protection physics; he is generally credited as being the father of modern electron and proton Monte Carlo methods. The methods he developed and the cross section data for which he was largely responsible are imbedded in nearly all of today’s coupled photon/charged-particle Monte Carlo codes. The use of electron-photon Monte Carlo calculations are now widespread, and the field has matured so much that scientists in many disciplines now rely heavily on the results of the better-known Monte Carlo codes rather than much more difficult measurements. Perhaps the most telling compliment is that his early pioneering work is still remarkably relevant today, more than 40 years later” [30].

### 5.2 Data Collections

NIST publishes standard reference data extensively. Several examples of early reports by RPD staff for x rays, gamma rays, and electrons were “X ray Attenuation Coefficients” by Gladys White in 1952 [31], “X ray Attenuation Coefficients from 10 keV to 100 MeV” by Gladys White Grodstein in 1957 [32], and “The X and Gamma Ray Energy Absorption or Transfer Coefficient: Tabulations and Discussion” by Rosemary T. Berger in 1961 [33].

An article by John Hubbell published in 1969 provided an evaluation and overall summary of previous works and was entitled “Photon cross sections, attenuation coefficients, and energy absorption coefficients from 10 keV to 100 GeV” [34]. This much-cited article reviewed the principal processes by which photons interact with matter. Other Hubbell reports are in Refs. [35–37].

### 5.3 Absorbed Dose Dosimetry

Steve Domen, formerly of the NBS Radiation Physics Division and now a NIST Guest Researcher, developed a sealed water calorimeter over a twenty year period [17,18,38–41], variations of which have been adopted internationally as primary measurement standards for absorbed dose. His 1994 article was entitled “A Sealed Water Calorimeter for Measuring Absorbed Dose” [17]. The calorimeter was a 305 mm (one foot) cube of water and thermistors for measuring temperature rise. The small thermistors were in specially processed water sealed in a small glass vessel positioned at a depth of 5 centimeters below the surface into which the gamma rays from a cobalt 60 source entered. A dose produced by the source of 1 Gy (100 rads) resulted in a measured temperature rise of 0.24 mK with a standard uncertainty of ±0.5 % (*k* = 1).

### 5.4 Radiation Spectrometry

The results illustrated in Fig. 4 for the continuous energy spectrum of x rays from a synchrotron measured with scintillation spectrometers having poor energy resolution demonstrated the inaccuracies in sorting out radiation effects produced by x rays at a single energy. As suggested above, the solution for the study of many nuclear and electromagnetic phenomena is to work with monoenergetic electrons that can be accurately controlled by magnets. For example, “from elastic scattering studies comes information about nuclear sizes and shapes, and from inelastic scattering comes information about the energy level structure of nuclei, including the spin, parity, and the transition strength of the excited nuclear states” [24]. The magnetic spectrometer system designed by Penner, Lightbody, and Fivozinsky and described in the two reports cited previously made such scattering studies possible [25,26]. “The energy resolution of the whole instrument was 0.08 percent — sufficient for high quality work” [24].

### 5.5 Photon Science

This research area is the one of the five categories that focused on the radiation, e.g., photons or electrons, rather than the targets of the radiation, e. g., atoms, nuclei or protection barriers. Experiments of William Dodge and Evans Hayward examined the theory and experiment of bremsstrahlung, of real and virtual photon interactions with nuclei, and of elastic scattering of photons [42–48]. Particularly elegant was the theory of Arenhoevel and Hayward on scattering of plane-polarized photons by the giant resonances of nuclei [42]. The equally elegant experimental application of this theory was described in an article by Hayward, Barber, and Sazama [43] in which “a beam of plane-polarized monochromatic photons” from a carbon target in a Linac bremsstrahlung beam was “produced by the resonance fluorescence of the well-known 1^+^ state at 15.1 MeV in ^12^C. These photons were scattered a second time from natural targets of Cd, Sn, Ta, W, Pt, Au, and Bi” [43]. This was indeed a brute force method of avoiding the problem caused by x ray sources with broad energy spectra in order to study nuclear phenomena at a single x ray/gamma ray energy, in this case 15.1 MeV.

All of the contributions and applications with the Linac and other Gaithersburg facilities described here from 1962 up to 1988 were made possible by the initial congressional hearings in 1959, at which the goals for the Linac envisioned by NBS Director Allen Astin were stated. The goals were “the assembly of basic data and measurements that make such applications possible ………” [49]. With goals considered to have been achieved, the Linac was shut down and disassembled in January 1989.

The termination of the work with the Linac at the end of time Period 3 is an appropriate place to end these “Recollections” and to discuss developments that led to the formation of CIRMS.

## 6. Radiation Measurements Today and the Need for CIRMS

The radiation industry today is quite different from the industry made possible by electron accelerators with low intensities. One significant difference is in the size of the units of measurement. Consider them first by making reference to the evolution of radiation measurements during much of the time period covered by these “Recollections”.

The International System of Units, universally abbreviated SI, is the modern metric system of measurement. The SI was established in 1960 by the Treaty of the Meter and the 11th General Conference on Weights and Measures (CGPM), an intergovernmental treaty organization. In the process of developing “coherent” quantities and units for ionizing radiation, the unit gray (Gy), where 1 Gy = 100 rad, was defined and agreed upon by CGPM in 1975. This unit became useful to users of radiation sources with high radiation intensities, because even the rad, where 1 rad = 100 ergs per gram, as used in work on the radiation protection of personnel had too small a magnitude as did the earlier unit, the erg per gram. Now users find it convenient to use an even larger unit, the kilogray, kGy.

Based on this up-grade in unit magnitudes, consider the absorbed dose requirements for processing of various materials and effects in the low dose range from 0.1 kGy to 3.0 kGy as shown in Table 2 and in the high dose range from 15 kGy to 1500 kGy in Table 3. These data were provided by Marshall Cleland in 2004 [50]. The list of processes and effects in these two tables help define those of interest to industry today.

Another change in the processing industry is the concern about inducing side-effect radioactivities in the materials being processed by the intense irradiation needed to accomplish the primary effects listed in Tables 2 and 3. As shown in a 1965 article by Koch and Eisenhower entitled “Radioactivity Criteria for Radiation Processing of Foods” [51], the radioactivities can be minimized by keeping the x ray, gamma ray, and electron radiation used for processing to energies below 10 MeV.

A final change in the industry is due to the recent developments of high-power, high-energy electron accelerators with peak energies below 10 MeV that were not available during the “Recollections” time periods. That situation has definitely changed with the present ready availability of commercial accelerators.

In summary, a measurement and standards world much more challenging than that described in these “Recollections” exists today principally because of the three factors just cited: the increasing doses required to produce certain effects up to 1000 kGy and beyond, the concern about radioactivities being induced in irradiated materials at electron beam energies above 10 MeV, and the availability of high-dose levels from new 100 kilowatt electron accelerators with peak energies less than 10 MeV.

Because of at least these challenges, the radiation processing industry agreed at the time the National Bureau of Standards became the National Institute of Standards and Technology in 1989 that they wanted improved coupling with NIST in work with ionizing radiations.

## 7. Formation of CIRMS in 1992

Under the leadership of Randy Caswell (then Chief of the Ionizing Radiation Division) [Appendix A], a mechanism for coupling NIST more closely than before with this industry and the public in general was established in 1992 with the formation of a Council on Ionizing Radiation Measurements and Standards (CIRMS) [52].

“CIRMS is an independent non-profit council that annually draws together experts involved in all aspects of ionizing radiation to discuss, review and assess developments and needs in this field” [52]. Its success derives from the interaction between people with needs for measurements and standards and those that satisfy those needs. The interaction is provided by the annual meeting comprising presentations and workshops and a triannual issuance of a Measurement Needs Report prepared by a Science and Technology Committee. The first of such reports was published in 1995 [52].

## Figures and Tables

**Table 1:** Radiation Facilities in NBS Radiation Physics Laboratory at Gaithersburg (1966)

140 MeV linear electron accelerator (Linac)
4 MeV electron Van de Graaff
3 MV positive ion Van de Graaff
1.5 MeV electron Dynamitron
500 kV constant-potential accelerator
Five x ray machines with maximum potentials of 50kV to 250 kV
180 MeV electron synchrotron

**Table 2:** Absorbed radiation doses (kGy) required to produce certain effects at doses 0.1 to 3.0 kGy

Sprout inhibition	0.1 – 0.2 kGy
Insect disinfestation	0.3 – 0.5 kGy
Parasite control	0.3 – 0.5 kGy
Delay of ripening	0.5 – 1.0 kGy
Fungi control	1.5 – 3.0 kGy
Bacteria control	1.5 – 3.0 kGy

**Table 3:** Absorbed radiation doses (kGy) required to produce certain effects at doses 15 to >>1500 kGy

Sterilization	15 – 30 kGy
Polymerization	25 – 50 kGy
Grafting	25 – 50 kGy
Crosslinking	50 – 150 kGy
Degradation	500 – 1500 kGy
Gemstone coloration	>> 1500 kGy

## Footnotes

2 The term “ionizing radiation” currently used by NIST in the names of organizational units provides better specificity than did the term “radiation” used earlier by NBS for its names.

3 “While at Princeton, he coauthored the Frank-Condon principle in molecular physics; developed the theory of radioactivity decay, with Ronald W. Gurney; a theory of optical rotary power; the theory of proton-proton scattering, with Gregory Breit; and the theory of charge independence of nuclear forces, with B. Cassen. His definitive treatise on the theory of atomic spectra, with George H. Shortley, established his reputation as an outstanding theoretical physicist.” [8]

4 “In 1937, Dr. Condon went to the Westinghouse Electric Corp. at Pittsburgh as associate director of research and there developed a program of nuclear research. Appointed a consultant to the National Defense Research Committee in 1940, he helped organize the Radiation Laboratory at MIT, where America’s microwave radar program was started, and wrote a basic textbook on the subject of microwaves for the laboratory. During the war he introduced and directed the microwave radar research program at Westinghouse.” [8]

5 “………In April 1943 he went to Los Alamos at the request of General Groves as associate director under Dr. Oppenheimer. Later that year he was called to the Radiation Laboratory at the University of California to head the theoretical physics group working on the electromagnetic (mass spectrograph) separation of uranium isotopes”. Toward the end of the war he started the nuclear reactor program at Westinghouse, which later produced the power plant for the Navy’s atomic submarine. [8]

6 Certain commercial equipment, instruments, or materials are identified in this paper to foster understanding. Such identification does not imply recommendation or endorsement by the National Institute of Standards and Technology, nor does it imply that the materials or equipment identified are necessarily the best available for the purpose.

## 8. Appendix A – NBS and NIST Leadership Assignments [1], [2] (NBS Radiation Physics Division and its Precursor, the Optics Division, 1928–1994)

In 1928, Lauriston Taylor was hired by Paul D. Foote, Chief of the Atomic Physics, Radium, X rays Section of the Optics Division. Foote was replaced in the April 1, 1930 staff directory by Fred L. Mohler as Section Chief. The July 1, 1945 NBS directory lists a new Section named X rays with Taylor as Section Chief. The March 1, 1950 directory lists a new Atomic and Radiation Physics Division with Robert D. Huntoon as Division Chief with an Atomic Physics Laboratory headed by Huntoon and a Radiation Physics Laboratory headed by Taylor with a Betatron Section with William Koch as Chief. Although the name, Radiation Physics Laboratory, was originally used for an organizational unit by NBS Director Edward U. Condon in the late 1940s, it was used also in the 1950s, but dropped in the December 1, 1960 directory. It was used in this article in the title of the 1966 booklet to designate the organizational unit housed in building 245 at Gaithersburg, Maryland.

At the beginning of 1962, Taylor was Chief of the Radiation Physics Division, Koch was Chief of the High Energy Radiation Section, and Randall Caswell was Chief of the Neutron Physics Section. In 1962, Taylor was made NBS Associate Director for Technical Support; Koch was appointed Chief of the Radiation Physics Division. In December 1964, Taylor retired from NBS. In February 1965, Koch continued as Chief of the Radiation Physics Division and also of the Nuclear Physics Branch of the Division. Joseph Motz was Chief of the Radiological Physics Branch, and James Leiss was Chief of the Accelerator Branch. In December 1966, Koch left NBS to accept the position of Director of the American Institute of Physics. Caswell was appointed as Acting Chief of the Radiation Physics Division. Subsequent reorganizations brought several Reactor Radiation Division groups together with parts of the Radiation Physics Division in December 1967 to form a Center for Radiation Research with Carl Muehlhause as Acting Director. Caswell was Acting Deputy Director. Leiss was Chief of the Linac Radiation Division and Motz was Chief of the Applied Radiation Division. In 1985, after several other reorganizations, the Center was abolished and parts of the 1966 Radiation Physics Division were relocated in an Ionizing Radiation Division under Caswell. Caswell retired in 1994 as Division Chief and was succeeded by Bert Coursey.

## 9. Appendix B – NCRP Reports

Complete Listing of Reports published by the National Council of Radiation Protection from 1931 to 1989 with Report Number, Title, and Year of Publication [9].

1. X Ray Protection (1931).
2. Radium Protection (1934).
3. X Ray Protection (1936).
4. Radium Protection (1938).
5. Safe Handling of Radioactive Luminous Compounds (1941).
6. Medical X Ray Protection up to Two Million Volts (1949).
7. Safe Handling of Radioactive Isotopes (1949).
8. Control and Removal of Radioactive Contamination in Laboratories (1951).
9. Recommendations for Waste Disposal of Phosphorus-32 and Iodine-131 for Medical Users (1951).
10. Radiological Monitoring Methods and Instruments (1952).
11. Maximum Permissible Amounts of Radioisotopes in the Human Body and Maximum Permissible Concentrations in Air and Water (1953).
12. Recommendations for the Disposal of Carbon-14 Wastes (1953).
13. Protection Against Radiation from Radium, Cobalt-60 and Cesium-137 (1954).
14. Protection Against Betatron-Synchrotron Radiations up to 100 Million Electron Volts (1954).
15. Safe Handling of Cadavers Containing Radioactive Isotopes (1953).
16. Radioactive Waste Disposal in the Ocean (1954).
17. Permissible Dose from External Sources of Ionizing Radiation (1954).
18. X Ray Protection (1955).
19. Regulation of Radiation Exposure by Legislative Means (1955).
20. Protection Against Neutron Radiation up to 30 Million Electron Volts (1957).
21. Safe Handling of Bodies Containing Radioactive Isotopes (1958).
22. Maximum Permissible Body Burdens and Maximum Permissible Concentrations of Radionuclides in Air and in Water for Occupational Exposure (1959).
23. Measurement of Neutron Flux and Spectra for Physical and Biological Applications (1960).
24. Protection Against Radiations from Sealed Gamma Sources (1960).
25. Measurement of Absorbed Dose of Neutrons and Mixtures of Neutrons and Gamma Rays (1961).
26. Medical X Ray Protection Up to Three Million Volts (1961).
27. Stopping Powers for Use With Cavity Chambers (1961).
28. A Manual of Radioactivity Procedures (1961).
29. Exposure to Radiation in an Emergency.
30. Safe Handling of Radioactive Materials (1964).
31. Shielding for High Energy Electron Accelerator Installations (1964).
32. Radiation Protection in Educational Institutions (1966).
33. Medical X Ray and Gamma Ray Protection for Energies up to 10 MeV - Equipment Design and Use (1968).
34. Medical X Ray and Gamma Ray Protection for Energies up to 10 MeV - Structural Shielding Design and Evaluation (1970).
35. Dental X Ray Protection (1970).
36. Radiation Protection in Veterinary Medicine (1970).
37. Precautions in the Management of Patients Who Have Received Therapeutic Amounts of Radionuclides (1970).
38. Protection Against Neutron Radiation (1971).
39. Basic Radiation Protection Criteria (1971).
40. Protection Against Radiation from Brachytherapy Sources (1972).
41. Specification of Gamma Ray Brachytherapy Sources (1974).
42. Radiological Factors Affecting Decision-Making in a Nuclear Attack (1974).
43. Review of the Current State of Radiation Protection Philosophy (1975).
44. Krypton-85 in the Atmosphere Accumulation, Biological Significance, and Control Technology (1975).
45. Natural Background Radiation in the United States (1975).
46. Alpha-Emitting Particles in Lungs (1975).
47. Tritium Measurement Techniques (1976).
48. Radiation Protection for Medical and Allied Health Personnel (1976).
49. Structural Shielding Design and Evaluation for Medical Use of X Rays and Gamma Rays of Energies up to 10 MeV (1976).
50. Environmental Radiation Measurements (1976).
51. Radiation Protection Design Guidelines for 0.1–100 MeV Particle Accelerator Facilities (1977).
52. Cesium-137 from the Environment to Man: Metabolism and Dose (1977).
53. Review of NCRP Radiation Dose Limit for Embryo and Fetus in Occupationally Exposed Women (1977).
54. Medical Radiation Exposure of Pregnant and Potentially Pregnant Women (1977).
55. Protection of the Thyroid Gland in the Event of Releases of Radioiodine (1977).
56. Radiation Exposure from Consumer Products and Miscellaneous Sources (1977).
57. Instrumentation and Monitoring Methods for Radiation Protection (1978).
58. A Handbook of Radioactivity Measurements Procedures (1978).
59. Operational Radiation Safety Program (1979).
60. Physical, Chemical and Biological Properties of Radiocerium Relevant to Radiation Protection Guidelines (1979).
61. Radiation Safety Training Criteria for Industrial Radiography (1978).
62. Tritium in the Environment (1979).
63. Tritium and Other Radionuclide Labeled Organic Compounds Incorporated in Genetic Material (1979).
64. Influence of Dose and Its Distribution in Time on Dose-Response Relationships for low-LET Radiations (1980).
65. Management of Persons Accidentally Contaminated with Radionuclides (1980).
66. Mammography (1980).
67. Radiofrequency Electromagnetic Fields Properties, Quantities and Units, Biophysical Interaction and Measurements (1981).
68. Radiation Protection in Pediatric Radiology (1981).
69. Dosimetry of X Ray and Gamma Ray Beams for Radiation Therapy in the Energy Range 10 keV to 50 MeV (1981).
70. Nuclear Medicine Factors Influencing the Choice and Use of Radionuclides in Diagnosis and Therapy (1982).
71. Operational Radiation Safety Training (1983).
72. Radiation Protection and Measurement for Low-Voltage Neutron Generators (1983).
73. Protection in Nuclear Medicine and Ultrasound Diagnostic Procedures in Children (1983).
74. Biological Effects of Ultrasound: Mechanisms and Clinical Implications (1983).
75. Iodine-129: Evaluation of Release from Nuclear Power Generation (1983).
76. Radiological Assessment: Predicting the Transport, Bioaccumulation, and Uptake by Man of Radionuclides Released to the Environment (1984).
77. Exposures from the Uranium Series with Emphasis on Radon and Its Daughters (1984).
78. Evaluation of Occupational and Environmental Exposures to Radon and Radon Daughters in the United States (1984).
79. Neutron Contamination from Medical Electron Accelerators (1984).
80. Induction of Thyroid Cancer by Ionizing Radiation (1985).
81. Carbon-14 in the Environment (1985).
82. SI Units in Radiation Protection and Measurements (1985).
83. The Experimental Basis for Absorbed-Dose Calculations in Medical Uses of Radionuclides (1985).
84. General Concepts for the Dosimetry of Internally Deposited Radionuclides (1985).
85. Mammography - A User’s Guide (1986).
86. Biological Effects and Exposure Criteria for Radiofrequency Electromagnetic Fields (1986).
87. Use of Bioassay Procedures for Assessment of Internal Radionuclide Deposition (1987).
88. Radiation Alarms and Access Control Systems (1986).
89. Genetic Effects from Internally Deposited Radionuclides (1987).
90. Neptunium: Radiation Protection Guidelines (1988).
91. Recommendations on Limits for Exposure to Ionizing Radiation (1987).
92. Public Radiation Exposure from Nuclear Power Generation in the United States (1987).
93. Ionizing Radiation Exposure of the Population of the United States (1987).
94. Exposure of the Population in the United States and Canada from Natural Background Radiation (1987).
95. Radiation Exposure of the U.S. Population from Consumer Products and Miscellaneous Sources (1987).
96. Comparative Carcinogenicity of Ionizing Radiation and Chemicals (1989).
97. Measurement of Radon and Radon Daughters in Air (1988).
98. Guidance on Radiation Received in Space Activities (1989).
99. Quality Assurance for Diagnostic Imaging (1988).
100. Exposure of the U.S. Population from Diagnostic Medical Radiation (1989).
101. Exposure of the U. S. Population from Occupational Radiation (1989).
102. Medical X Ray, Electron Beam and Gamma Ray Protection for Energies up to 50 MeV (Equipment Design, Performance and Use) (1989).
